# Endothelial dysfunction due to eNOS uncoupling: molecular mechanisms as potential therapeutic targets

**DOI:** 10.1186/s11658-023-00423-2

**Published:** 2023-03-09

**Authors:** Anna Janaszak-Jasiecka, Agata Płoska, Joanna M. Wierońska, Lawrence W. Dobrucki, Leszek Kalinowski

**Affiliations:** 1 Department of Medical Laboratory Diagnostics - Fahrenheit Biobank BBMRI.Pl, Medical University of Gdansk, 7 Debinki Street, 80-211, Gdansk, Poland; 2 Department of Neurobiology, Polish Academy of Sciences, Maj Institute of Pharmacology, 12 Smętna Street, 31-343 Kraków, Poland; 3 Department of Bioengineering, University of Illinois at Urbana-Champaign, Urbana, IL USA; 4 Beckman Institute for Advanced Science and Technology, 405 N Mathews Ave, MC-251, Urbana, IL 61801 USA; 5 Department of Biomedical and Translational Sciences, Carle-Illinois College of Medicine, Urbana, IL USA; 6 BioTechMed Centre, Department of Mechanics of Materials and Structures, Gdansk University of Technology, 11/12 Gabriela Narutowicza Street, 80-233 Gdansk, Poland

**Keywords:** Cardiovascular disease, Endothelial dysfunction, eNOS uncoupling, Oxidative/nitroxidative stress, Peroxynitrite, Nitric oxide, ADMA, Tetrahydrobiopterin, BH_4_

## Abstract

Nitric oxide (NO) is one of the most important molecules released by endothelial cells, and its antiatherogenic properties support cardiovascular homeostasis. Diminished NO bioavailability is a common hallmark of endothelial dysfunction underlying the pathogenesis of the cardiovascular disease. Vascular NO is synthesized by endothelial nitric oxide synthase (eNOS) from the substrate L-arginine (L-Arg), with tetrahydrobiopterin (BH_4_) as an essential cofactor. Cardiovascular risk factors such as diabetes, dyslipidemia, hypertension, aging, or smoking increase vascular oxidative stress that strongly affects eNOS activity and leads to eNOS uncoupling. Uncoupled eNOS produces superoxide anion (O_2_^−^) instead of NO, thus becoming a source of harmful free radicals exacerbating the oxidative stress further. eNOS uncoupling is thought to be one of the major underlying causes of endothelial dysfunction observed in the pathogenesis of vascular diseases. Here, we discuss the main mechanisms of eNOS uncoupling, including oxidative depletion of the critical eNOS cofactor BH_4_, deficiency of eNOS substrate L-Arg, or accumulation of its analog asymmetrical dimethylarginine (ADMA), and eNOS S-glutathionylation. Moreover, potential therapeutic approaches that prevent eNOS uncoupling by improving cofactor availability, restoration of L-Arg/ADMA ratio, or modulation of eNOS S-glutathionylation are briefly outlined.

## Introduction

Cardiovascular disease (CVD) is the leading cause of death worldwide, highlighting the need to investigate its molecular mechanisms for effective treatment options. A common and early hallmark of CVD is endothelial dysfunction, i.e., a disturbance in the normal physiology of the endothelium that lines all blood vessels [1]. The endothelium is critical to cardiovascular homeostasis and plays a vital role in the pathophysiology of cardiovascular diseases associated with atherosclerosis, including hypertension, stroke, coronary artery disease, peripheral vascular disease, or heart failure [2, 3]. Endothelial cells produce and release a subset of diverse signaling molecules that orchestrate cardiovascular physiology by regulating hemostasis, vascular tone and permeability, inflammation, and angiogenesis [4]. Among these substances, nitric oxide (NO) is a key molecule that significantly influences the physiology of the endothelium and the cardiovascular system, and endothelial dysfunction is often simply defined as diminished NO bioavailability [5].

Nitric oxide was discovered as an endothelium-derived relaxing factor (EDRF), and research into its essential role in vascular tone regulation has been recognized by the Nobel Prize awarded to Furchgott, Ignarro, and Murad in 1998 [6]. NO, as a lipophilic molecule, easily diffuses from endothelium into adjacent vascular smooth muscle cells (VSMCs) and binds to the prosthetic haem group of soluble guanylate cyclase (sGC), thus activating the enzyme [7]. sGC catalyzes the dephosphorylation of guanosine triphosphate (GTP) to cyclic guanosine 3’,5’-monophosphate (cGMP), which acts as a second messenger and activates protein kinase G (PKG) [8]. As a result of PKG activity, cytoplasmic calcium (Ca^2+^) levels decrease, and the downstream signaling cascade leads to vascular smooth muscle relaxation and consequent vasodilation [9]. But regulating vascular tone is not the only role of NO as it also regulates vascular wall permeability, reduces proliferation and migration of VSMCs as well as platelet activation and aggregation [10]. Moreover, NO modulates the expression of endothelial adhesion molecules and thus prevents leukocyte recruitment and adhesion [11]. Therefore, besides being a potent vasodilator, NO generally has anti-atherosclerotic properties. Its pleiotropic effects are critical for vascular homeostasis, and dysregulation of NO signaling pathways is associated with the pathogenesis of CVD [12]. Endothelial dysfunction is characterized by impaired endothelium-dependent vasorelaxation due to diminished nitric oxide bioavailability resulting from an imbalance between its generation and degradation.

In endothelial cells, NO is produced by endothelial nitric oxide synthase (eNOS), one of three nitric oxide synthases (NOS) present in human tissues besides neuronal NOS (nNOS) expressed primarily in neurons and inducible NOS (iNOS) expressed in various cell types (especially in immune system cells) during infection or inflammation [13]. Thus, in addition to its essential role in the regulation of vascular physiology, NO also functions as a neurotransmitter in the nervous system and as a cytotoxic agent in the immune response [14, 15]. Given the very short NO half-life, its molecular effects are restricted to the site of its synthesis, hence eNOS expressed almost exclusively in endothelial cells is the major donor of vascular NO. Thus, diminished eNOS expression and activity are the major causes of reduced NO synthesis. On the other hand, NO scavenging by superoxide anion (O_2_^−^) is the main reason for decreased NO half-life in the vasculature [16]. Therefore it is not surprising that increased oxidative stress is involved in the pathogenesis of CVD [17].

A unique phenomenon called eNOS uncoupling combines oxidative NO scavenging with altered eNOS activity. Uncoupled eNOS generates highly reactive superoxide (O_2_^−^) instead of NO. eNOS uncoupling is often triggered by oxidative stress associated with cardiovascular risk factors, including diabetes, hypertension, dyslipidemia, smoking, and aging, via the mechanisms described below [18]. The presence of both functional and uncoupled eNOS in the cell results in the concomitant production of NO and O_2_^−^ in the close vicinity. O_2_^−^ reacts with NO, thus scavenging it and yielding harmful peroxynitrite radical (ONOO^−^), so a vicious cycle arises that potentiates oxidative stress and drives pathological changes [19]. Moreover, eNOS uncoupling occurs to some extent physiologically, as we have shown by using high-precision electrochemical microsensors able for concomitant real-time detection of NO, O_2_^−^ and ONOO^−^ generated in a single cell [20–22]. eNOS uncoupling is thought to be one of the major underlying causes of endothelial dysfunction observed in the pathogenesis of vascular diseases. The presence of uncoupled eNOS has been proven in patients with diabetes [23–25], hypertension [26], coronary artery disease [27], and congestive heart failure [28]. eNOS uncoupling was also demonstrated in animal experimental models of hypertension [29], diabetes [30], ischemia–reperfusion injury [31, 32], and ageing [33], and confirmed in vitro in human and animal endothelial cell cultures [34, 35].

This review discusses the molecular mechanisms leading to eNOS uncoupling and the proposed therapeutic approaches to prevent or reverse eNOS uncoupling and restore endothelial function as a possible strategy for CVD treatment.

## eNOS regulation

**Fig. 1:**
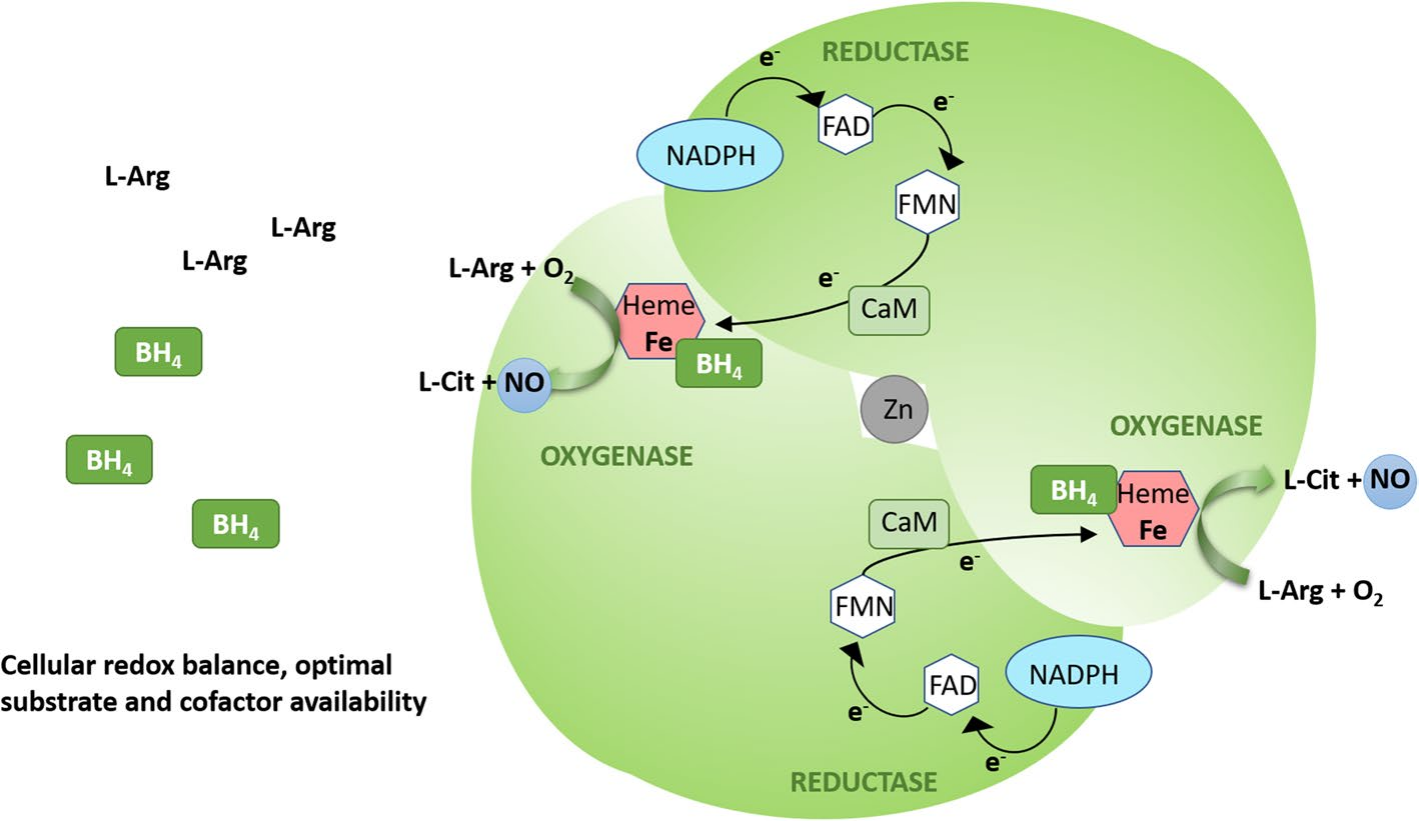
Schematic eNOS homodimer structure with two monomers orientated "head-to-tail". Each monomer consists of a C-terminal reductase domain that binds NADPH, FAD, and FMN, a central calmodulin-binding region, and an N-terminal oxygenase domain that binds substrate L-Arg, oxygen, heme, and BH_4_. The formation of a homodimer enables the transfer of electrons from the reductase domain of one monomer to the oxygenase domain of the second monomer. The dimeric structure is stabilized by heme binding and by zinc ion in the zinc-thiolate cluster at the dimer interface. During catalysis, electrons from NADPH flow through the flavins FAD and FMN to the heme of the opposite monomer and CaM increases the rate of interdomain electron transfer. Heme reduction enables O_2_ binding, and BH_4_ can donate an electron to reduce and activate O_2_. When cellular redox balance is maintained, and the substrate L-Arg and the essential cofactor BH_4_ availabilities are optimal, O_2_ reduction is coupled to L-Arg oxidation and NO synthesis. L-Cit is formed as a byproduct

NO is produced by eNOS from L-Arg with molecular oxygen (O_2_) and nicotinamide adenine dinucleotide phosphate (NADPH) as co-substrates. The reaction requires several cofactors: heme, flavin adenine dinucleotide (FAD), flavin mononucleotide (FMN), and tetrahydrobiopterin (BH_4_), the latter being a critical determinant of eNOS coupling [13]. eNOS activity is also dependent on Ca^2+^/calmodulin (CaM) binding [36]. The enzyme exists as a homodimer, each monomer consisting of an N-terminal oxygenase domain with substrate L-Arg, heme, zinc, and BH_4_ cofactor binding sites, a central CaM-binding region, and a C-terminal reductase domain with NADPH, FAD, and FMN binding sites (Fig. 1) [37]. Functional eNOS dimer catalyzes the electron transfer from the C-terminal-bound NADPH through FAD and FMN of one monomer to the heme iron in the N-terminal oxygenase domain of the second monomer, and this interdomain electron transfer is facilitated by CaM. Heme iron is reduced, thus enabling the binding and reduction of O_2_. BH_4_ serves in this process as a one-electron donor for the heme-bound oxygen, which activates O_2_, enabling the following oxidation of L-Arg to L-citrulline (L-Cit) and NO (Fig. 1) [38–40]. The BH_4_ cofactor is thus essential for optimal eNOS activity [41].

eNOS is expressed constitutively in endothelial cells and basal NO synthesis maintains resting vascular tone, however a number of factors dynamically influence the enzyme expression and activity [13, 42, 43]. eNOS is regulated by various stimuli through post-translational modifications: phosphorylation, acetylation, S-nitrosylation, S-glutathionylation, and protein–protein interactions [44, 45]. Palmitoylation and myristoylation of eNOS enable its localization to the plasmalemmal caveolae, where the enzyme is sequestered in its inactive state due to the interaction with caveolin-1 [46]. eNOS activity is dependent on intracellular Ca^2+^ concentration. In response to acetylcholine or bradykinin Ca^2+^ level increases, and Ca^2+^-activated CaM binds eNOS, disrupts its inhibitory interaction with caveolin, and stimulates NO synthesis [47]. Growth factors, hormones and shear stress affects the activity of kinases (PKA, Akt, and AMPK) and phosphatases, and modulate eNOS activity by altering the phosphorylation status of the enzyme [48]. Phosphorylation at Ser1177, Ser633 and Ser615 stimulates eNOS, whereas phosphorylation at Thr495 and Ser114 inhibits it [49]. Among the various post-translational modifications of eNOS, S-glutathionylation is of particular interest to this review as it may directly affect eNOS uncoupling [50], thus we will discuss this issue in more detail below.

## Oxidative/nitroxidative stress in cardiovascular diseases

Reactive oxygen species (ROS) are produced as by-products of cellular metabolism and play an important role in physiological cell signaling [51, 52]. ROS can also contribute to the pathogenesis of various diseases, especially if their amount exceeds the capacity of the antioxidant defense system, causing oxidative stress and subsequent oxidative damage to lipids, proteins, and DNA [53]. Oxidative stress is a hallmark of CVD [54]. It is well documented that cardiovascular risk factors such as dyslipidemia, diabetes, hypertension, obesity, or smoking lead to increased production of ROS in the vascular wall, and the resulting oxidative stress promotes the development of endothelial dysfunction [55]. Oxidative stress is also a major cause of ischemia–reperfusion injury, observed after the restoration of blood flow to the ischemic tissue following myocardial infarction or stroke [56, 57].

Enzymes that produce free radicals in the vascular wall include NADPH oxidase, xanthine oxidase, the mitochondrial electron transport chain, and, importantly, uncoupled eNOS [58]. Superoxide generated by these enzymes can be reduced by superoxide dismutase (SOD) to H_2_O_2_ which is then eliminated by catalase and glutathione peroxidase (GPx). However, superoxide can also interact with NO and inactivate it, yielding toxic ONOO^−^, and the rate of this reaction is three times faster than the dismutation of O_2_^−^ by SOD (6.7 × 10^9^ mol/L^−1^ s^−1^) [59]. Thus the balance between antioxidant defense enzymes and the amount of ROS generated is key to proper NO bioavailability since excessive O_2_^−^ scavenges NO. This balance is altered in various pathological conditions resulting in oxidative stress that plays an essential role in the development of CVD [60–62].

NADPH oxidases are considered as the major producers of O_2_^−^ in the vasculature, and moreover, they are regarded the main source of “kindling radicals” that trigger the activation of additional ROS sources, e.g., via eNOS uncoupling [63]. The expression and activity of NADPH oxidases have been documented to increase in experimental models of diabetes [64, 65], hypertension [66, 67], smoking [68], obesity [69, 70], and with ageing [71]. Increased expression or activity of NADPH oxidase was reported in coronary and peripheral arteries of patients with coronary artery disease [72–74]. It is now well established that activation of NADPH oxidases contributes to cardiovascular pathogenesis [75].

NADPH oxidase- generated O_2_^−^, besides NO scavenging, triggers eNOS uncoupling (the mechanisms are discussed in the next section), and uncoupled eNOS becomes itself a source of superoxide [19]. The reaction of superoxide with NO yields peroxynitrite, ONOO^−^, a very potent oxidant that intensifies eNOS uncoupling by oxidizing its cofactor BH_4_ [76]. Moreover, ONOO^−^ cause protein oxidation and nitration, leading to cellular injury [77]. Thus, uncoupled eNOS contributes significantly to vascular oxidative stress, and acting as a vicious cycle, it is considered to be one of the most important mechanisms leading to endothelial dysfunction. eNOS uncoupling has been reported in the vessels of patients with diabetes, hypertension, coronary artery disease [23–27] as well as in animal studies [29–31, 33].

Another source of free radicals that contributes to endothelial dysfunction is the electron transport chain and oxidative phosphorylation. Elevated levels of plasma glucose and free fatty acids increase mitochondrial superoxide production [78]. Excessive ROS production at the mitochondrial compartment is associated with cardiovascular diseases and has been observed in diabetes, aging, hypertension, and heart failure [79–82].

Also, xanthine oxidase-derived ROS contributes to endothelial dysfunction. The enzyme activation has been implicated in increased vascular O_2_^−^ generation in patients with chronic heart failure [83], coronary artery disease [72, 84] and in the animal model of hypercholesterolemia [85]. It was reported that the expression and activity of xanthine oxidase in endothelial cells is upregulated by angiotensin II (Ang II) treatment [86]. Xanthine oxidase is also involved in endothelial dysfunction induced by smoking, since its inhibition restored endothelial function in heavy smokers [87].

According to the “kindling radical” hypothesis, individual sources of free radicals are interrelated and can stimulate each other, which is confirmed by the observations that inhibiting only one of them can restore the redox balance [88]. NADPH oxidase, xanthine oxidase and mitochondria-derived ROS, in addition to damaging cellular proteins, lipids, and DNA, can scavenge NO, and moreover, they can induce eNOS uncoupling [18]. Uncoupled eNOS not only does not produce NO but instead generates O_2_^−^ exacerbating oxidative stress. In addition to NO scavenging and eNOS uncoupling, vascular oxidative stress induces oxidative damage of cellular macromolecules and the expression of proinflammatory genes, thus promoting atherogenesis [58, 89]. Oxidative stress is a major contributor to eNOS uncoupling and its mechanisms are described below.

## Mechanisms of eNOS uncoupling

Under physiological conditions, i.e. in normal eNOS activity, the interdomain electron transfer and NADPH oxidation are coupled to NO synthesis. eNOS uncoupling refers to a situation in which eNOS produces superoxide instead of NO, thus becoming a source of harmful free radicals rather than antiatherosclerotic NO [18, 19].. Conditions implicated in eNOS uncoupling include oxidative depletion of the critical eNOS cofactor BH_4_, deficiency of eNOS substrate L-Arg, or accumulation of its analog asymmetrical dimethylarginine, and eNOS S-glutathionylation. These individual mechanisms are discussed below, but it is worth noting here that they are not mutually exclusive and may occur simultaneously.

### Deficiency of BH_4_ cofactor

BH_4_ is an essential eNOS cofactor required for efficient electron transfer in the eNOS catalytic cycle that largely determines its activity [90]. Cellular production of BH_4_ is dependent on two alternative pathways: de novo synthesis or regeneration from its oxidized form dihydrobiopterin (BH_2_) through the salvage pathway [91]. BH_4_ is synthesized de novo from GTP by guanosine triphosphate cyclohydrolase I (GTPCH), 6-pyruvoyltetrahydropterin synthase (PTPS) and sepiapterin reductase (SR) and GTPCH is the rate-limiting enzyme in BH_4_ biosynthesis [91]. Importantly, under oxidative stress conditions, BH_4_ is rapidly oxidized to BH_2_ by superoxide anion or, especially strongly, by peroxynitrite derived from NO scavenging by O_2_^−^ [76]. BH_2_ can be reduced back to BH_4_ via the salvage pathway by dihydrofolate reductase (DHFR) [92]. Thus, the cellular availability of BH_4_ is dependent on cellular redox status and the level of expression and activity of GTPCH and DHFR, the latter enzyme being particularly essential under oxidative stress conditions.

**Fig. 2:**
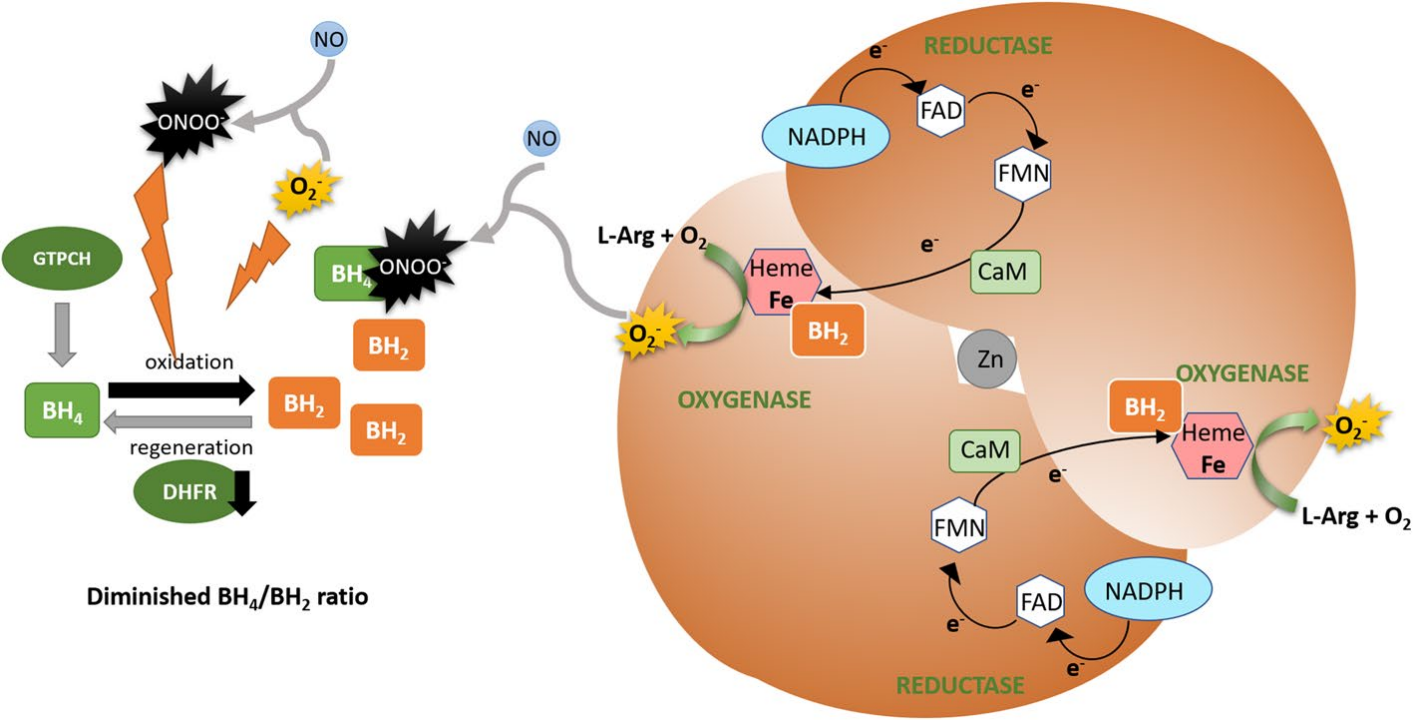
eNOS uncoupling due to BH_4_ deficiency. Under conditions of oxidative stress, O_2_^−^ can combine with NO yielding ONOO^−^, which strongly oxidizes BH_4_ to BH_2_. Decreased DHFR expression or activity prevents effective regeneration of the cofactor. BH_2_ competes with BH_4_ at the heme oxygenase domain but is not catalitically active, thus disturbing the normal electron flow and promoting superoxide formation

Cardiovascular risk factors are associated with oxidative stress, and excessive O_2_^−^ oxidizes BH_4_ to BH_2_ [93]. BH_2_ can competitively replace BH_4_, but being catalytically incompetent as a cofactor, it promotes eNOS uncoupling, where electron transport is uncoupled from NO synthesis, and instead, O_2_^−^ is generated (Fig. 2) [90, 94]. Moreover, the peroxynitrite formed from the reaction of O_2_^−^ with NO very strongly oxidizes BH_4_ [76]. As a result, the cellular BH_4_/BH_2_ ratio drops, further increasing eNOS uncoupling and driving a vicious cycle of oxidative stress.

The suboptimal concentration of BH_4_ and more importantly the resulting decrease in the BH_4_/BH_2_ ratio probably represent a major cause of eNOS uncoupling implicated in the pathophysiology of endothelial dysfunction [95, 96]. Oxidative depletion of BH_4_ as a cause of eNOS uncoupling and endothelial dysfunction has been described in vivo, in the aortas of mice with deoxycorticosterone acetate-salt (DOCA-salt) hypertension [29], spontaneously hypertensive mice and rats [97, 98], apolipoprotein E (poE)-deficient mice [99], or aged mice and rats [33, 100]. Human studies have also confirmed the relationship between BH_4_ depletion and endothelial dysfunction. Decreased vascular BH_4_ level, increased production of eNOS-dependent O_2_^−^ proving eNOS uncoupling, and impaired vasorelaxations in response to acetylcholine were reported in patients with coronary artery disease [101]. Depletion of BH_4_ and reduced NO bioavailability were also shown in patients with peripheral arterial disease [102], diabetes [103], hypertension [26], and hypercholesterolemia [104]. Decreased BH_4_/BH_2_ ratio is also associated with endothelial dysfunction in heart failure with preserved ejection fraction (HFpEF) patients [105].

Importantly, the reduction of the BH_4_/BH_2_ ratio is not only a consequence of oxidative depletion of BH_4_, but may also a result from the reduced synthesis and regeneration of the cofactor due to decreased expression or activity of GTPCH and DHFR under oxidative stress conditions. Reduced GTPCH expression with a concomitant decrease in NO levels was observed in the aortas of aged mice, whereas GTPCH overexpression restored proper endothelial function [106]. Diabetes significantly affects the BH_4_/BH_2_ ratio in mouse aortas without changing the total biopterin level or GTPCH expression, which indicates that BH_4_ oxidation is the main cause of its deficiency [107]. Slightly different conclusions can be drawn from the studies on the diabetic rat model, where BH_4_ deficiency was shown to be due to decreased expression and activity of GTPCH [108]. Nevertheless, in both cases, the overproduction of GTPCH improved the BH_4_/BH_2_ ratio and restored endothelial function [107, 109]. Diabetes is also associated with impaired cofactor regeneration. Decreased DHFR expression and BH_4_ content along with increased eNOS-derived O_2_^−^ were observed in aortas of streptozotocin (STZ)-induced diabetic mice model [110]. Accordingly, decreased DHFR expression, accumulation of BH_2_, decreased BH_4_/BH_2_ ratio, and eNOS uncoupling were observed in vitro, in hyperglycemic endothelial cells [96, 111]. Oxidative stress evoked by exposure of endothelial cells to Ang II in vitro resulted in downregulation of DHFR expression, decrease in BH_4_ levels, and eNOS uncoupling [112]. Decreased DHFR expression is also involved in eNOS uncoupling and vascular disorders in hypertensive rats [97] and in hypercholesterolemic mice [113]. The decreased BH_4_/BH_2_ ratio observed in various cardiovascular pathologies is therefore due to not only oxidative depletion of BH_4_, but also impaired synthesis and regeneration of this cofactor.

### Deficiency of substrate L-Arg and accumulation of ADMA

**Fig. 3:**
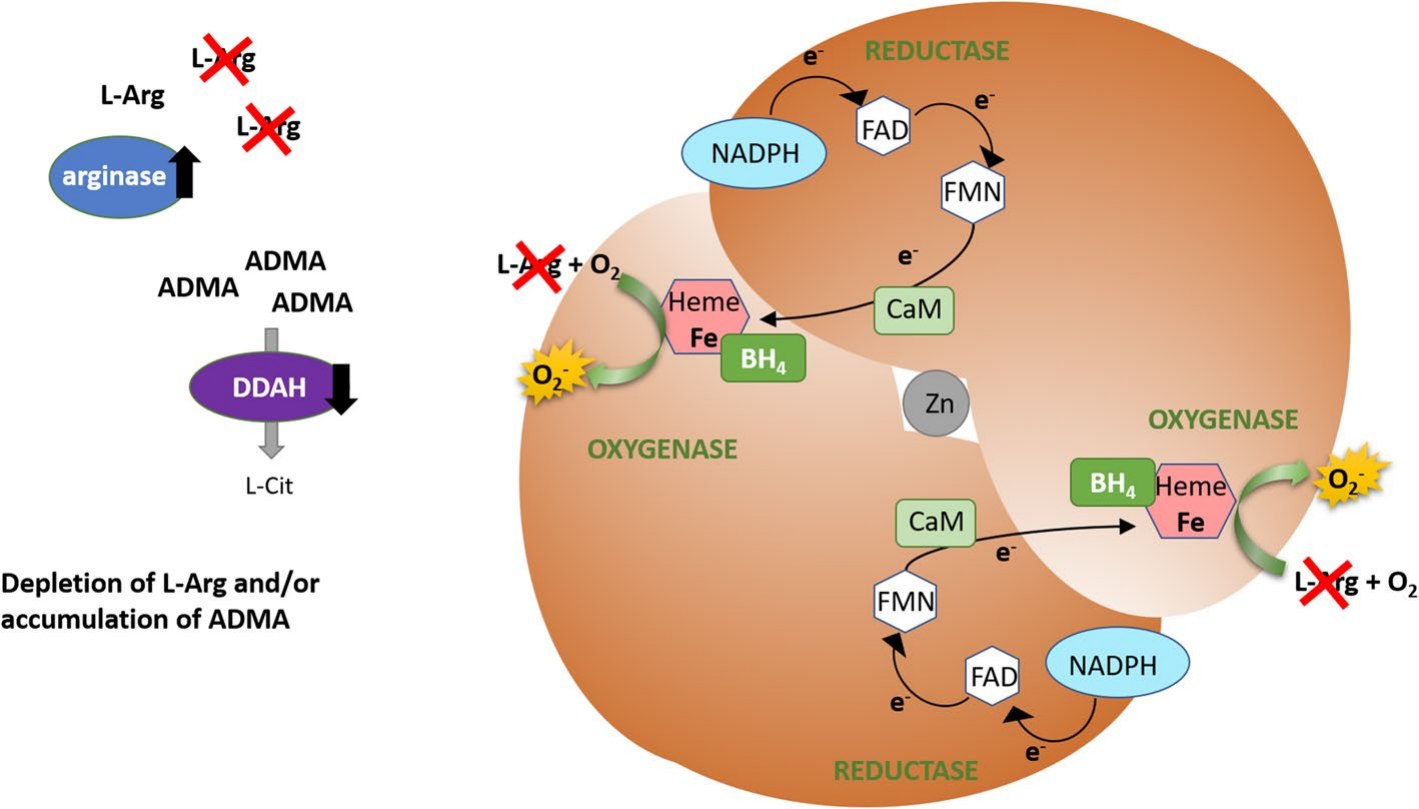
eNOS uncoupling due to diminished L-Arg/ADMA ratio. Under reduced L-Arg availability (resulting from excessive arginase activity) and/or accumulation of ADMA (due to decreased DDAH activity), the substrate concentration may not be sufficient to saturate eNOS and/or L-Arg is outcompeted by ADMA. As a result, molecular oxygen is a final electron acceptor, leading to superoxide formation

L-Arg is the substrate for eNOS and the main precursor of NO, therefore, the availability of L-Arg is important for the activity of this enzyme, and the substrate insufficiency may lead to eNOS uncoupling (Fig. 3). The cellular content of this amino acid is dependent on dietary intake, whole-body protein turnover, endogenous synthesis, cellular uptake, and metabolism [114, 115]. Under physiological conditions, the intracellular concentration of L-Arg is saturating, as it significantly exceeds the K_m_ of eNOS [116]. Nevertheless, exogenous L-Arg can still stimulate NO synthesis, which is a phenomenon known as the "L-arginine paradox" [117]. Therefore, the effective concentration of L-Arg, particularly the ratio of L-Arg to its methylated derivative, the asymmetric dimethylarginine (ADMA), an inhibitor of eNOS, is essential, and decreased L-Arg/ADMA ratio is associated with eNOS uncoupling [118]. The efficiency of cellular L-Arg uptake and the rate of its intracellular metabolism may also play a role and affect the final availability of L-Arg for eNOS. L-Arg is transported across the endothelial cell membrane mainly by cationic amino acid transporter (CAT) proteins belonging to the Na^+^-independent y + transport system (the letter y is for lysine, the first substrate described for this system, and the + denotes the positive charge of CAT substrates) [119]. The major endothelial L-Arg transporter, cationic amino acid transporter 1 (CAT-1), colocalizes with eNOS in plasma-membrane caveolae and could directly deliver L-Arg to eNOS or increase its local concentration in eNOS proximity [120, 121]. Moreover, the intracellular concentration of L-Arg is modulated by arginase- an enzyme that hydrolyzes L-Arg to ornithine and urea and competes with eNOS for a common substrate [122]. There are two isoforms of arginases in humans, with Arg-I being particularly important in the hepatic urea cycle and Arg-II being distributed throughout various tissues, especially kidneys [123]. Both isoforms have been reported to be expressed in endothelium, although their expression seems to be species and vascular bed–specific, e.g., both Arg-I and Arg-II are present in human aortic endothelial cells (HAECs), whereas in human umbilical vein endothelial cells (HUVECs), Arg-I is barely detectable [124, 125].

Cardiovascular risk factors are associated with diminished L-Arg availability [126]. The significance of L-Arg availability is emphasized by the beneficial effects of L-Arg supplementation on endothelial function and cardiovascular health, which will be discussed in more detail in the next section. Both L-Arg uptake and metabolism can be altered in CVD pathophysiology. It was shown that homocysteine-induced oxidative stress significantly decreased CAT-1 expression in endothelial cells, resulting in inhibition of L-Arg uptake, reduced NO production, and increased ONOO^−^ formation, indicating eNOS uncoupling, which was abolished by L-Arg supplementation [127]. Still, the regulation of L-Arg transport by cardiovascular risk factors is not well understood. In turn, the role of arginases and its impact on L-Arg availability and CVD pathophysiology associated with oxidative stress is much better known and seems to be of great importance.

Arginases are involved in the pathogenesis of age-related diseases, including CVD, as aging affects their expression and activity [128]. In old rats and mice compared to young, arginase activity was significantly increased and accompanied by decreased NO production and increased O_2_^−^ generation indicating eNOS uncoupling [129, 130]. Arginase inhibition or silencing significantly reduced eNOS-derived O_2_^−^ level and restored eNOS coupling and endothelial function [129, 130]. In humans, the expression of Arg-I and Arg-II in the vascular wall was demonstrated to enhance with age and obesity, concomitantly with increased vascular superoxide and diminished NO levels [131]. Arginase is also involved in diabetes-induced vascular dysfunction. Diabetic patients showed increased expression of Arg-I and decreased NO production in coronary arterioles, resulting in reduced vasodilation that could be restored by arginase inhibition or L-Arg application [132]. Increased Arg-I expression and activity, and increased superoxide generation were also reported in aortas and liver of STZ-diabetic rats, which showed decreased NO-mediated vasodilation in coronary vessels, that could be restored by arginase inhibition [133]. Similarly, exposure of bovine coronary endothelial cells to high glucose concentrations resulted in increased Arg-I expression and activity and diminished NO levels, whereas silencing of Arg-I restored NO production [133]. Arginase plays also a crucial role in the pathophysiology of cholesterol-mediated endothelial dysfunction. Endothelial arginase is activated in atherogenic-prone apoE-deficient mice as well as in wild-type mice fed a high-cholesterol diet [134]. Inhibition or deletion of Arg-II prevents a diet-dependent decrease in NO production and increase in ROS production in the vessels, restores endothelial function, and prevents atherogenesis [134]. Similar results were obtained in vitro; oxidized low-density lipoprotein (LDL) activated Arg-II in HAECs, leading to impaired NO production [135, 136]. Arginase activation, decreased NO, increased ROS resulting in endothelial dysfunction and vascular stiffness were also observed in wild-type mice exposed to cigarette smoke, in contrast to Arg-II knockout mice, suggesting that Arg-II contributes to smoking-induced vascular dysfunction [137].

Most of the plasmatic and cellular L-Arg comes from physiological whole-body protein turnover [114]. However, L-Arg residues within proteins are commonly subjected to methylation carried-out post-translationally by a family of nine enzymes named protein arginine methyltransferases (PRMTs 1–9) [138]. Therefore, the subsequent breakdown of such proteins results in the release of methylated arginine derivatives: NG-monomethyl-L-arginine (L-NMMA), asymmetric dimethylarginine (ADMA), and symmetric dimethylarginine (SDMA) [139]. Methylarginines released from the protein breakdown into the cytosol pass into the bloodstream, and can be taken up by other cells via y + transporters, thus they can interfere with L-Arg uptake [140, 141]. Moreover, when taken up by endothelial cells, both ADMA and L-NMMA, but not SDMA, compete with L-Arg for eNOS binding but are not active as substrates, thus leading to eNOS uncoupling. ADMA is considered the most potent endogenous inhibitor of eNOS [142]. While under physiological conditions, ADMA plasma concentration fluctuates in the range of 1–2 µM, it increases significantly (up to tenfold) in the presence of oxidative stress associated with cardiovascular risk factors [143]. Increased ADMA levels in plasma have been correlated with endothelial dysfunction and are an independent risk factor for the development of systemic cardiovascular diseases [142].

The circulating ADMA is partially eliminated by the kidneys, but the most part is metabolized to L-Cit and dimethylamine by dimethylarginine dimethylaminohydrolases (DDAHs) that are expressed in two isoforms [144]. DDAH-1 is responsible for the systemic elimination of circulating ADMA, and its expression is most pronounced in the liver, kidneys, brain, and lungs, however it is also found in the endothelium. DDAH-2 role in ADMA metabolism seems to be more local, and DDAH-2 is expressed primarily in blood vessels, heart, placenta, and immune tissues [144]. Impaired DDAH activity is associated with ADMA accumulation observed in diverse clinical conditions [143]. Hence, plasma and intracellular ADMA levels result from its generation and metabolism regulated by PRMTs and DDAHs, respectively.

Oxidative stress affects the expression and activity of these enzymes, giving rise to ADMA accumulation, and a resulting decrease in L-Arg/ADMA ratio, i.e., diminished effective substrate availability, leads to eNOS uncoupling (Fig. 3). Elevated plasma ADMA levels and impaired endothelium-dependent vasodilation were observed in patients with hypercholesterolemia, hyperhomocysteinemia, diabetes, and hypertension [118, 145–148]. It was proved that oxidized LDL cholesterol enhances the expression of PRMTs, decreases DDAH activity, and increases the release of ADMA from human endothelial cells in vitro [149, 150]. Oxidized LDL was also demonstrated to decrease DDAH activity in hypercholesterolemic rabbits [150]. The activity of aortic DDAH was reduced, and plasma ADMA levels were increased in STZ-diabetic rats [151]. Consistently, in human endothelial cells exposed to high glucose, the activity of DDAH was significantly impaired with concomitant ADMA accumulation and reduction of cGMP level, indicating impaired eNOS activity that could be reversed by antioxidant treatment [151].

### eNOS S-glutathionylation

As mentioned earlier, eNOS can be S-glutathionylated. In this type of post-translational modification, the tripeptide glutathione composed of glycine, cysteine, and glutamate is linked by a disulfide to specific cysteine residues of a protein [152]. Glutathione is produced ubiquitously in eukaryotes, and its intracellular concentration is in the millimolar range [153]. The reduced form of glutathione (GSH) is considered the essential non-enzymatic antioxidant in the body and the first line of defense against oxidants since it scavenges free radicals, undergoing oxidation to disulfide GSSG [154]. The ratio of reduced to oxidized glutathione (GSH/GSSG) is a marker of cellular health. Under physiological conditions, this ratio exceeds 100; thus, GSH constitutes over 99% of the cellular glutathione pool. Under pathological states associated with redox imbalance and oxidative stress, this ratio drops, and altered glutathione redox status (GSH/GSSG) increases protein S-glutathionylation by direct disulfide exchange between thiol protein and GSSG. On the other hand, ROS oxidizes protein thiols to sulfenic acid, which can be reduced by S-glutathionylation with GSH [155].

It is believed that S-glutathionylation is a regulatory mechanism that protects proteins from irreversible oxidation of sulfhydryl groups by oxidative stress to sulfinic and sulfonic acids resulting in protein degradation [155]. The consequence of such protection may be a change in the activity of the proteins, their oligomerization status, or the ability to interact with their ligands or protein partners, which is not always advantageous. Reversible protein S-glutathionylation can be thus considered as a redox switch that regulates cellular function under oxidative stress by modulating the activity of metabolic and signaling enzymes [156].

**Fig. 4:**
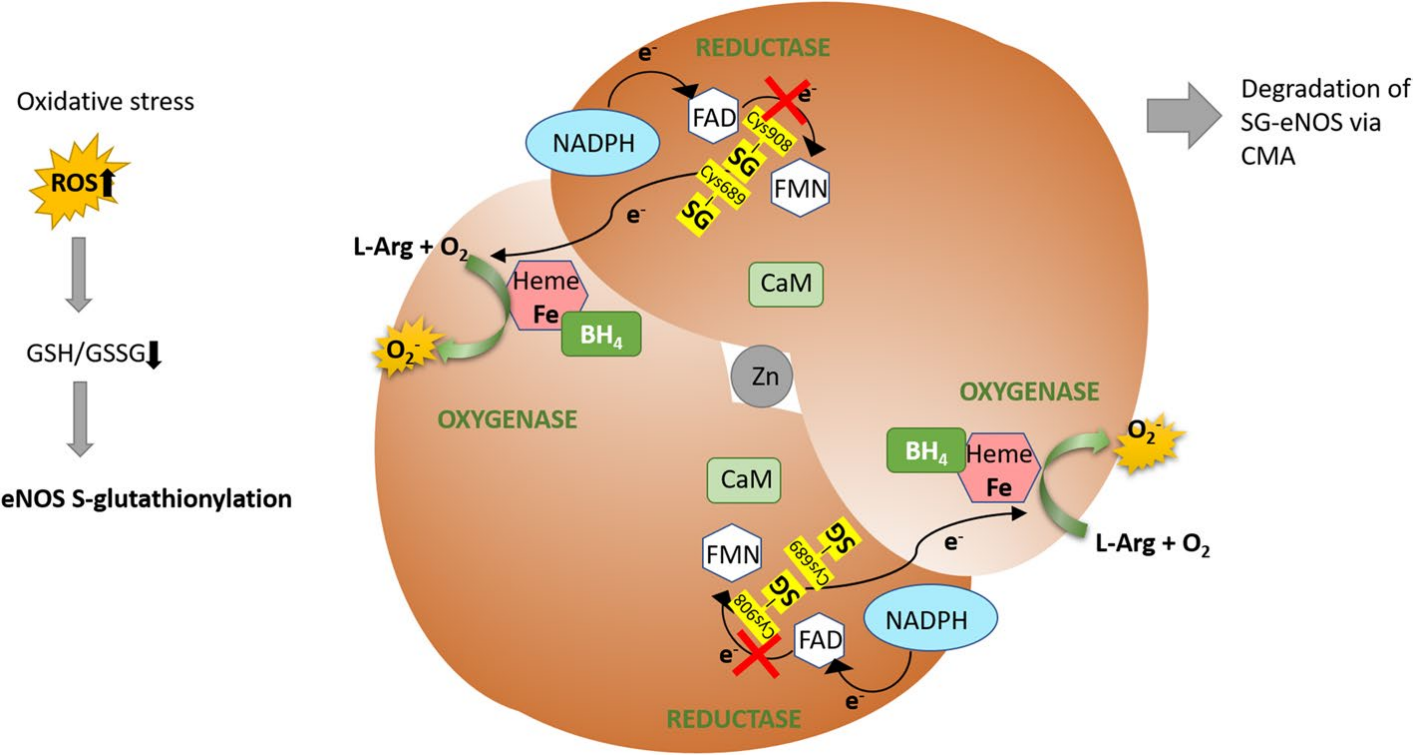
eNOS uncoupling due to eNOS S-glutathionylation. Oxidative stress decreases the cellular GSH/GSSG ratio, leading to protein S-glutathionylation. Glutathionylated cysteine residues (Cys689 and Cys908) of eNOS are located at the interface of the FAD and FMN binding sites, thus disrupting FAD-FMN alignment and electron transfer between flavins, which causes the transfer of an electron to molecular oxygen and the production of a superoxide radical instead of NO. Prolonged retention of S-glutathionylated eNOS (SG-eNOS) in the cytoplasm can result in its degradation via chaperone-mediated autophagy (CMA), leading to irreversible loss of eNOS

Importantly, eNOS activity can undergo such redox regulation. Chen et al. [50] reported that S-glutathionylation uncouples eNOS thus changing its activity and function. Oxidized glutathione was shown to dose-dependently induce S-glutathionylation of two conserved cysteine residues (Cys689 and Cys908) in the eNOS reductase domain, resulting in eNOS uncoupling characterized by a decreased NO production and an increased generation of O_2_^−^ [50, 157, 158]. The proposed mechanism by which S-glutathionylation uncouples eNOS assumes that the glutathione binding alters protein structure. Both modified cysteine residues are located at the interface of the FAD- and FMN- binding sites, thus S-glutathionylation would disrupt FAD-FMN alignment and electron transfer between these flavins, resulting in the transfer of an electron to molecular oxygen and the production of a superoxide radical instead of NO (Fig. 4). S-glutathionylation-induced eNOS uncoupling mechanism is unique since O_2_^−^ is generated in the reductase domain and is not inhibited by N(ω)-nitro-L-arginine methyl ester (L-NAME). In contrast, uncoupling mechanisms dependent on substrate and cofactor availability occur primarily at the heme of the oxygenase domain and can be blocked by L-NAME [50, 159].

The influence of S-glutathionylation-dependent eNOS uncoupling on endothelium function was tested on isolated rat vessels. Aortic segments exposed to glutathione reductase inhibitor, 1,3-bis(2-chloroethyl)-1-nitrosourea (BCNU) showed markedly decreased endothelium-dependent vasodilation. Moreover, the involvement of this mechanism in the pathogenesis of cardiovascular diseases was confirmed in vivo. High levels of S-glutathionylated eNOS and impaired endothelium-dependent vasodilation were demonstrated in the vessels of spontaneously hypertensive rats; in contrast, control normotensive rats presented low eNOS S-glutathionylation levels and proper vasodilation response [50].

Ang II, the principal effector of the renin-angiotensin system linked to the pathogenesis of several CVD, was demonstrated to increase eNOS S-glutathionylation via NADPH oxidase activation in cultured endothelial cells, as well as in human arteries ex vivo [160]. Moreover, attenuation of Ang II signaling reduced the level of eNOS glutathionylation and improved endothelium-dependent vasorelaxation in vivo in rabbits [160]. S-glutathionylation-dependent eNOS uncoupling can also contribute to the pathophysiology of preeclampsia (PE) in humans since a high level of eNOS S-glutathionylation was detected in PE placentas in contrast to control placentas of healthy patients [161]. The significance of that mechanism was also confirmed in the pathophysiology of diabetes-related endothelial dysfunction by demonstrating the presence of S-glutathionylated eNOS in the aortas of STZ-induced diabetic rats [162]. Furthermore, eNOS uncoupling due to its S-glutathionylation was observed in endothelial cells in response to hypoxia-reoxygenation [35], and eNOS S-glutathionylation was confirmed in vivo in the coronary arteries of murine myocardial ischemia–reperfusion injury model [163]. Moreover, it was demonstrated that if S-glutathionylated eNOS is not deglutathionylated and persists in the cytosol, it is degraded via chaperone-mediated autophagy, resulting in irreversible loss of uncoupled eNOS, which protects cells from continuous production of O_2_^−^ [164].

Interestingly, two mechanisms of eNOS uncoupling, i.e., the one dependent on BH_4_ depletion and that induced by the enzyme's S-glutathionylation, are interrelated. It was demonstrated that eNOS uncoupling induced by BH_4_ deficiency stimulates eNOS S-glutathionylation [165, 166]. O_2_^−^ generated from uncoupled eNOS oxidizes eNOS cysteine 908 thiol residue, forming a protein thiyl radical susceptible to S-glutathionylation by GSH [166]. On the other hand, S-glutathionylation of eNOS induces BH_4_ deficiency. It was shown that inhibition of glutathione reductase in endothelial cells induces a fivefold increase in eNOS S-glutathionylation, and the resulting eNOS uncoupling (shown by superoxide generation) leads to BH_4_ oxidation, BH_2_ accumulation, and decreased BH_4_/BH_2_ ratio. The two mechanisms of eNOS uncoupling, S-glutathionylation-induced and BH_4_-dependent, are functionally related, and moreover, their effects are additive [165].

## Pharmacological prevention of eNOS uncoupling

**Fig. 5:**
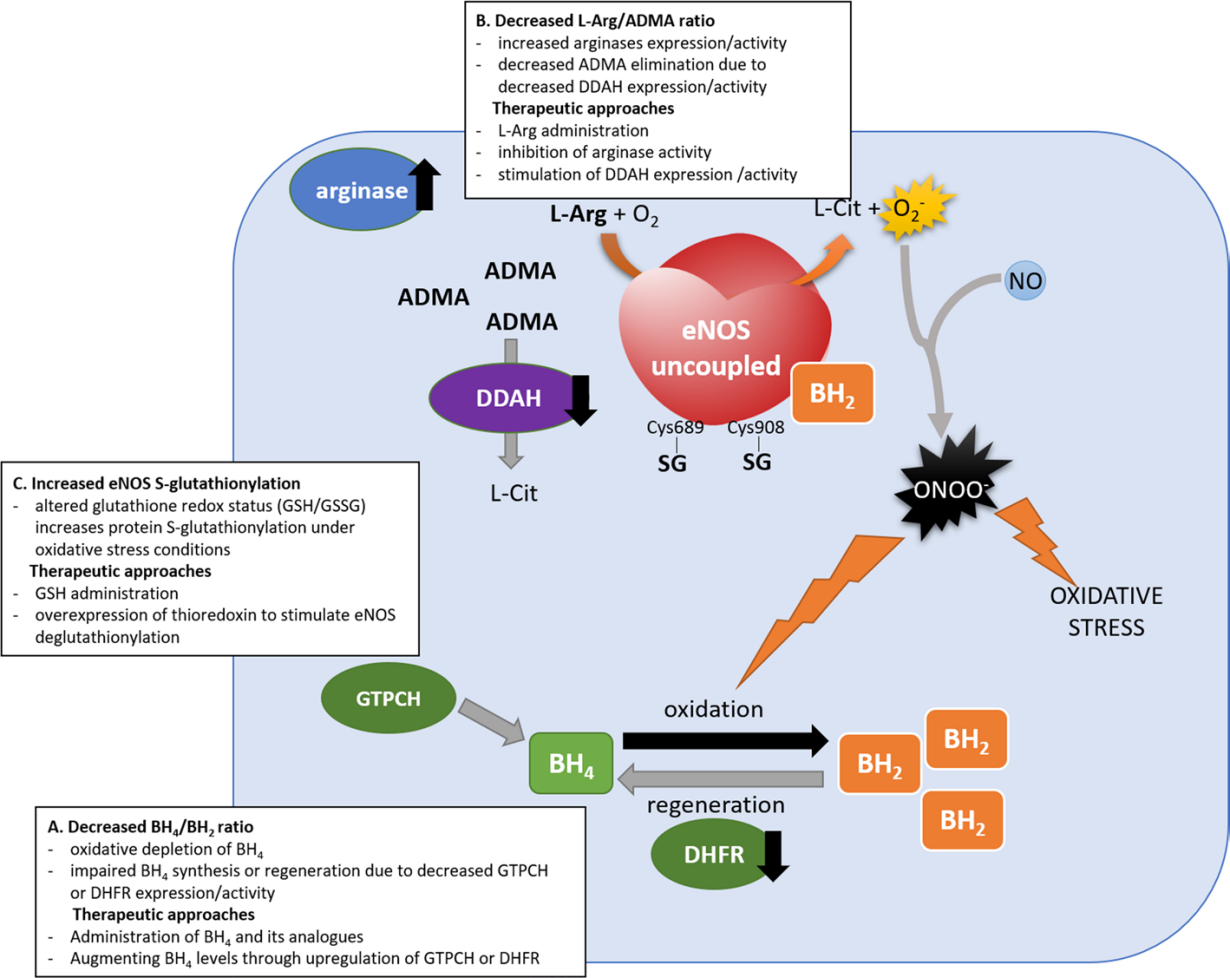
Major causes of eNOS uncoupling as targets of potential therapeutic interventions. Oxidative stress associated with cardiovascular risk factors leads to eNOS uncoupling by: **A** decreased BH_4_/BH_2_ ratio due to oxidation of BH_4_ and impairment of DHFR expression/activity; **B** decreased L-Arg/ADMA ratio due to excessive arginase expression/activity and diminished DDAH expression/activity; **C** eNOS S-glutathionylation at Cys689 and Cys908

Understanding the mechanisms and the importance of eNOS uncoupling as a one of major causes of endothelial dysfunction has made reversing or preventing eNOS uncoupling an attractive therapeutic approach to prevent or treat cardiovascular complications. Since eNOS can be both an NO and an O_2_^−^ producing enzyme, eNOS targeting may have a dual effect on vascular function, depending on its functional state. Thus, precise determination of eNOS activity and coupling state and the rate of eNOS-dependent NO and O_2_^−^ generation are of particular importance when assessing the effects of drugs on endothelial function. For example, using electrochemical ultramicrosensors, we have demonstrated that antihypertensive drugs cicletanine, nifedipine, and third-generation β-blockers (nebivolol, carvedilol), Ang II AT_1_ receptor antagonists, as well as statins, concurrently stimulate NO release and scavenge O_2_^−^ thus reducing the formation of ONOO^−^ and preventing endothelial dysfunction [167–172]. Similarly, we have also demonstrated the potential of endogenous nicotinamide metabolite N^1^-methylnicotinamide (MNA^+^) in the prevention of eNOS uncoupling [173]. Since excessive vascular ROS generation is the key driver of endothelial dysfunction and the main trigger of eNOS uncoupling, attempts have been made to mitigate oxidative stress. However, antioxidant strategies will not be discussed here since this broad topic has been excellently reviewed elsewhere [174–177]. Instead, we will focus on strategies aiming to prevent eNOS uncoupling by targeting the exact mechanisms of this phenomenon such as restoration of BH_4_/BH_2_ ratio, L-Arg/ADMA ratio, and physiological eNOS glutathionylation level (Fig. 5). Below, they are briefly outlined.

### Restoration of the BH_4_/BH_2_ ratio

Given the critical role of BH_4_ in eNOS activity and endothelial health, numerous interventions involving BH_4_ supplementation have been attempted to improve vascular function. The efficacy of BH_4_ treatment in preventing eNOS uncoupling was demonstrated in animal models [29, 98, 178] as well as in human studies. Acute, intravenous BH_4_ administration augmented endothelium-dependent vasodilation in hypertensive individuals [26], in patients with hypercholesterolemia [104, 179], coronary artery disease [180], heart failure [181], as well as in chronic smokers [182], or in healthy subjects [183, 184]. However, chronic (several weeks), oral BH_4_ administration gave slightly discrepant results. BH_4_ supplementation improved endothelial function in patients with hypercholesterolemia [185], hypertension [186], and rheumatoid arthritis [187]. In contrast, BH_4_ administration for several weeks in patients with coronary artery disease did not improve endothelial function, but instead increased BH_2_ levels [188]. BH_4_ is very unstable and is easily oxidized pterin. Oxidative stress associated with cardiovascular disease oxidizes BH_4_, so the administration of additional BH_4_ under such conditions may further decrease the BH_4_/BH_2_ ratio. Therefore it was proposed that coadministration of BH_4_ with antioxidants could be a better strategy to restore the proper BH_4_/BH_2_ ratio [189, 190].

Alternatively, a BH_4_ precursor sepiapterin can be administrated that is converted to BH_4_ via the salvage pathway, and it was shown to restore tissue BH_4_ levels even more efficiently that BH_4_ supplementation in mice [191]. Sepiapterin was demonstrated to improve vascular reactivity in animal models of diabetes [192] and obesity [193]. Human studies revealed that sepiapterin administration is able to restore coronary flow mediated dilation in diabetic patients [24]. However, studies on isolated vessels of hyperlipidemic rabbits have shown that although sepiapterin restored vascular BH_4_ levels, it impaired NO-dependent vasodilation [194]. High concentrations of sepiapterin may compete with BH_4_ for binding to eNOS and thus promote eNOS uncoupling [195]. In addition, the conversion of sepiapterin to BH_4_ requires DHFR activity, therefore, in states in which the activity or expression of DHFR is reduced, sepiapterin supplementation may be ineffective [196].

Folic acid and its active circulating form 5-methyltetrahydrofolate (5-MTHF) have been also show to increase vascular BH_4_/BH_2_ ratio, reverse eNOS uncoupling and restore endothelial function [27, 197]. First, folic acid improves metabolic homocysteine clearance, and hyperhomocysteinemia is a CVD risk factor [198]. However, the effects of folate supplementation on endothelial function are also homocysteine-independent [199]. Folates can directly interact with eNOS and improve the binding affinity of BH_4_ to eNOS, chemically stabilize BH_4_ and enhance the regeneration of BH_4_ from BH_2_ [199]. Human experimental studies have proven the effectiveness of folates in improving eNOS-dependent endothelial function. Low-dose oral folic acid treatment was sufficient to improve vascular function in patients with coronary artery disease [200]. Infusion of 5-MTHF improved the impaired endothelium-dependent vasodilation in patients with familial hypercholesterolemia [201], diabetes [25], and coronary artery disease [27]. In contrast, in patients with chronic heart failure, 5-MTHF infusion did not improve endothelial function; however, it significantly reduced serum ADMA concentrations, suggesting a direct effect of 5-MTHF on ADMA metabolism [202]. Although the experimental clinical data were promising, clinical trials on the use of folic acid in CVD treatment have produced conflicting results that mostly failed to prove the beneficial effect of folic acid supplementation on cardiovascular health. Meta-analysis of several clinical trials data indicated that folic acid supplementation is not effective for cardiovascular events prevention in people with pre-existing vascular disease [203]. Other meta‐analyses indicated a modest but significant benefit of folic acid supplementation for stroke prevention with more significant benefit observed among participants without preexisting CVD or with lower plasma folate levels at baseline [204, 205].

Interestingly, BH_4_ levels can be increased by statins. Treatment of human endothelial cells in vitro with fluvastatin and cerivastatin augmented GTPCH expression and BH_4_ levels [206, 207]. These effects seem to be at least partly mediated by microRNA. Lovastatin has been shown to inhibit aberrant miR-133a expression that targets GTPCH, thereby restoring BH_4_/BH_2_ ratio, contributing to eNOS recoupling and preventing endothelial dysfunction [208]. Beneficial influence of statins on vascular BH_4_ content was also observed in animal and human studies. In STZ-diabetic rats, atorvastatin administration increased GTPCH expression, thus preventing eNOS uncoupling [209]. Simvastatin increased GTPCH activity and BH_4_ production in hypertensive rats [210]. In patients with coronary artery disease, atorvastatin upregulated GTPCH expression, increased BH_4_ levels and improved vascular NO bioavailability [211]. Patients with multiple coronary risk factors treated with atorvastatin were reported to have increased plasma BH_4_/BH_2_ ratio and showed improved flow-mediated dilation [212].

### Restoration of L-Arg/ADMA ratio

L-Arg availability and its ratio to inhibitory ADMA is an important determinant of eNOS activity. Therefore, attempts have been made to restore L-Arg/ADMA ratio by direct L-Arg administration, inactivation of arginases or stimulation of ADMA elimination.

As mentioned earlier, many studies have reported a positive effect of L-Arg supplementation on eNOS activity (known as L-arginine paradox) in conditions associated with endothelial dysfunction such as dyslipidemia, diabetes, hypertension or coronary artery disease [213]. It is believed that L-Arg supply can prevent eNOS uncoupling through various mechanisms. Most of all, exogenous L-Arg supplementation can alter the L-Arg/ADMA ratio and thus overcome the inhibitory effects of ADMA on eNOS as well as on y + transporters [117]. Moreover, L-Arg itself acts as an antioxidant so that it can help to maintain a proper BH_4_/BH_2_ ratio [214]. Thus, L-Arg supplementation seemed to be an attractive therapeutic strategy for cardiovascular diseases, preventing eNOS uncoupling and increasing NO synthesis in the endothelium [215, 216]. However, the results of experimental clinical studies and clinical trials are contradictory. Most studies report a vasodilation effect induced by L-Arg administration. High doses of L-Arg administrated intravenously induced NO-dependent vasodilation in healthy subjects as well as in patients with peripheral arterial disease or coronary artery disease [217–220]. Oral L-Arg supplementation was shown to enhance endothelial function in patients with metabolic syndrome or with hyperhomocysteinemia [221, 222]. Meta-analysis of 11 randomized, double-blind, placebo-controlled trials involving 387 participants indicated that oral L-Arg administration significantly lowers blood pressure [223]. Since ADMA is endogenous inhibitor of eNOS and L-Arg/ADMA ratio determines eNOS activity [118], L-Arg supplementation is proposed to be particularly beneficial in patients with elevated ADMA levels, as supported by results of studies in animal models of hypercholesterolemia and arteriosclerosis [117]. Despite these promising results, optimism is not allowed due to studies that undermine the effectiveness of L-Arg supplementation and even show its harmfulness. Long-term administration of L-Arg in patients with peripheral arterial disease did not increased NO synthesis nor improved vascular reactivity [224]. In myocardial infarction therapy, L-Arg administration was not effective as it did not improve vascular stiffness measurements or ejection fraction and may be associated with higher post-infarction mortality [225]. The ineffectiveness of L-Arg supplementation, or even its harmfulness, may have several reasons. Cardiovascular diseases in which L-Arg supplementation therapy has been tested are associated with oxidative stress and oxidative depletion of the essential eNOS cofactor BH_4_, causing eNOS uncoupling. Thus, substrate supply without the additional amount of available cofactor may not be sufficient to reverse eNOS uncoupling. Moreover, high doses of L-Arg can induce the expression of arginases, which metabolizes L-Arg, and increase arginase activity is associated with eNOS uncoupling and endothelial dysfunction [122, 226, 227]. High levels of L-Arg have been also demonstrated to competitively inhibit DDAH activity, thus contributing to increased levels of ADMA which is endogenous inhibitor of eNOS [228].

Since elevated arginase expression and/or activity is associated with eNOS uncoupling, inhibition of arginase activity may be a good therapeutic strategy for endothelial dysfunction. To experimentally modulate arginase activity, specific inhibitors have been developed: N-hydroxy-L-arginine (NOHA) or N-hydroxy-nor-l-arginine (nor-NOHA), and boronic acid derivatives, such as 2(S)-amino-6-boronohexanoic acid (ABH), and S-(2-boronoethyl)-l-cysteine (BEC) s[229]. These inhibitors as well as genetically modified arginase knockout animals were tested in experimental studies which allowed to determine the effect of arginase on endothelial function and eNOS activity. Inhibition of arginase activity with BEC or deletion of Arg2 gene prevented eNOS uncoupling and atherogenesis in the vessels of hypercholesterolemic mice [134]. In mice with diet-induced obesity, deletion of Arg1 or Arg2 gene or inhibition of arginase activity with ABH prevented vascular dysfunction [230, 231]. Similarly, endothelial dysfunction in STZ-diabetic mice was reversed by ABH [232]. Also short-term clinical studies with local administration of arginase inhibitor have shown promising results. Intra-arterial infusion of the arginase inhibitor nor-NOHA for two hours significantly improved endothelium-dependent vasodilation in patients with familial hypercholesterolemia [233], coronary artery disease [234], diabetes [235, 236] and in elderly subjects [237]. However, the compounds used so far do not have specificity as to the enzyme isoform, and further studies are needed to search for novel and more specific arginase inhibitors [238]. It is also worth mentioning that increased arginase activity in diabetes is influenced by insulin administration, and insulin infusion has been shown to reduce arginase activity in patients with type 2 diabetes [239].

Moreover, excessive arginase activity can be mitigated with statins. Simvastatin and lovastatin blocked arginase activation by oxidized LDL in HAECs, and lovastatin prevented arginase activation in apoE-deficient mice fed a high-cholesterol diet [136]. In diabetic rats simvastatin diminished diabetes-induced arginase activity and Arg-I expression, reduced oxidative stress and restored proper vasorelaxation in response to acetylcholine [133]. Human studies demonstrated that atorvastatin decreased arginase activity in hypercholesterolemic patients [240].

Modulating the expression/activity of DDAH may also be a promising therapeutic strategy to restore the favorable L-Arg/ADMA ratio and several experimental studies have shown that the increase in DDAH expression reduces ADMA levels and stimulates NO synthesis. The use of purified recombinant DDAH-1 to lower ADMA levels was proved to be effective for the treatment of ischemia–reperfusion myocardial damage in isolated mouse hearts [241]. Some long-known medications can diminish ADMA levels by increasing DDAH expression. For example, nebivolol and telmisartan stimulated the expression of DDAH-2 in cultured endothelial cells, resulting in reduction of ADMA concentration. [242, 243]. Moreover, nebivolol treatment as well as telmisartan administration reduced serum ADMA levels and improved endothelial function in essential hypertensive patients [244, 245]. Also statins have been reported to increase ADMA metabolism by upregulation of DDAH. In cultured endothelial cells, simvastatin increased DDAH-1 expression and decreased ADMA content [246]. Rosuvastatin and atorvastatin increased DDAH expression and reduced serum ADMA levels in a rat model of pulmonary hypertension and in in high-fat diet-induced insulin-resistant rats with endothelial dysfunction, respectively [247, 248]. However, human studies have not produced conclusive results. Some clinical trials confirmed that statin treatment reduces circulating ADMA levels [249, 250], while others failed to prove such an effect [251, 252].

### Modulating eNOS S-glutathionylation

S-glutathionylation is a reversible post-translational modification, and a mixed disulfide bond between a protein cysteine residue and glutathione can be reduced back in a process of deglutathionylation. The cellular antioxidant systems of glutaredoxin and thioredoxin are able to reduce thiol groups and restore protein function [253]. It was demonstrated that glutaredoxin (Grx1), a cytosolic oxidoreductase, can efficiently deglutathionylate eNOS in the presence of GSH, i.e., when the cellular redox status is favorable [254]. However, under oxidative stress conditions, when GSSG level is increased, Grx1 glutathionylate eNOS. Therefore, Grx1 activity is influenced by GSH/GSSG ratio [254]. Since lowered GSH/GSSG ratio promotes eNOS S-glutathionylation and uncoupling, supplementation of GSH may be beneficial for endothelial function. It was demonstrated that GSH administration reverses endothelial dysfunction and improves NO bioavailability in atherosclerotic patients, possibly due to its general anti-oxidant properties [255]. However, GSH could likely stimulate eNOS deglutathionylation by Grx1 and thus recouple the enzyme.

Another pathway that allows deglutathionylation is independent of GSH and involves thioredoxin (Trx), a small, ubiquitous redox protein. The primary function of Trx is the reduction of oxidized cysteine groups on proteins [253]. Trx possesses the disulfide reductase activity in its reduced state, which is maintained by the thioredoxin reductase in a NADPH-dependent reaction [256]. Interestingly, Trx can deglutathionylate eNOS even in the presence of high levels of GSSG under oxidative stress conditions [163]. Trx overexpression prevented eNOS glutathionylation and uncoupling in vivo in coronary arteries of ischemia/reperfusion treated mice and protected against myocardial infarction. Accordingly, in human coronary artery endothelial cells (HCAECs) in vitro, Trx overexpression protected against hypoxia/reoxygenation-induced eNOS glutathionylation and preserved eNOS activity, whereas Trx silencing resulted in increased eNOS S-glutathionylation and uncoupling [163].

Therapeutic potential of Trx has been also noted in studies on age-related hypertension in mice. It was demonstrated that overexpression of human Trx in mice protected against endothelial dysfunction and prevented the development of age-related hypertension [257]. Moreover, injection of recombinant human Trx via tail vein into aged wild-type mice reversed the existing hypertension. Both overexpression of Trx in transgenic mice and injection of Trx into old wild-type mice improved endothelial-dependent relaxation [257]. In aged mice eNOS S-glutathionylation was increased and eNOS was uncoupled, producing reduced amounts of NO and being a major source of vascular O_2_^−^. In contrast, in aged transgenic mice overproducing Trx the level of eNOS S-glutathionylation, NO and eNOS-derived O_2_^−^ production were not significantly different from that of young mice [257]. These studies highlight the potential antihypertensive properties of Trx, which modulates the vascular redox state, prevents eNOS S-glutathionylation and preserves eNOS activity in the vessels of aged animals. Translational studies are needed to assess the potential of GSH, glutaredoxin and thioredoxin to modulate eNOS uncoupling and improve endothelial function in humans. Moreover, the efficiency of the eNOS deglutathionylation mechanisms affects the half-life of the eNOS protein, as it was demonstared in ischemia–reperfusion injury [164]. Persisting S-glutathionylation leads to the degradation of eNOS, thus protecting cells against oxidative damage, but on the other hand, it leads to irreversible loss of eNOS [164].

eNOS glutathionylation state could be also modified indirectly. Since Ang II increases eNOS S-glutathionylation and uncoupling, angiotensin-converting enzyme inhibition could reverse glutathionylation-dependent eNOS uncoupling [160]. Indeed, attenuation of Ang II signaling by captopril was demonstrated to reduce eNOS S-glutathionylation and endothelial O_2_^−^ generation, simultaneously increasing NO production and improving vasorelaxation in rabbits [160].

## Conclusions

It is now well recognized that eNOS uncoupling is associated with endothelial dysfunction and the pathophysiology of cardiovascular disease, thus, the mechanisms leading to eNOS uncoupling are considered a promising therapeutic target. However, direct interventions aimed at restoring eNOS cofactor and substrate availability, such as administration of BH_4_ analogs or supplementation of L-Arg, did not produce a clearly beneficial outcome. The results of arginase inhibition trials encourage further research to better understand the specificity and pharmacokinetics of new inhibitors. Further studies are also required to assess the therapeutic potential of GSH, Grx, and Trx in the treatment of endothelial dysfunction associated with eNOS S-glutathionylation. Moreover, already known drugs can modulate the eNOS coupling state. As mentioned above, statins were reported to restore favorable BH_4_/BH_2_ ratio and L-Arg/ADMA ratio through augmenting GTPCH expression, abolishing excessive arginase activity, or increasing DDAH activity. Thus it is worth emphasizing that the pleiotropic action of statins, among the many beneficial effects for endothelial physiology [258], also includes the prevention of eNOS uncoupling.

## Data Availability

Not applicable.

## Abbreviations

5-MTHF: 5-Methyltetrahydrofolate
ADMA: Asymmetrical dimethylarginine
Ang II: Angiotensin II
apoE: Apolipoprotein E
BH_2_: Dihydrobiopterin
BH_4_: Tetrahydrobiopterin
CaM: Calmodulin
cGMP: Cyclic guanosine monophosphate
CVD: Cardiovascular disease
DDAH: Dimethylaminohydrolase
DHFR: Dihydrofolate reductase
eNOS: Endothelial nitric oxide synthase
FAD: Flavin adenine dinucleotide
FMN: Flavin mononucleotide
GPx: Glutathione peroxidase
Grx1: Glutaredoxin
GSH: Glutathione
GSSG: Glutathione disulfide
GTP: Guanosine triphosphate
GTPCH: Guanosine triphosphate cyclohydrolase I
L-Arg: L-arginine
L-Cit: L-citrulline
LDL: Low-density lipoprotein
L-NAME: N(ω)-nitro-L-arginine methyl ester
NADPH: Nicotinamide adenine dinucleotide phosphate
NO: Nitric oxide
O_2_^−^: Superoxide anion
ONOO^−^: Peroxynitrite
PRMT: Protein arginine methyltransferase
ROS: Reactive oxygen species
sGC: Soluble guanylate cyclase
SOD: Superoxide dismutase
STZ: Streptozotocin
Trx: Thioredoxin
VSMCs: Vascular smooth muscle cells
